# Efficacy and safety of immunotherapy-based neoadjuvant regimens in locally advanced gastric cancer: a meta-analysis based on high-quality clinical trials

**DOI:** 10.1097/JS9.0000000000002815

**Published:** 2025-06-20

**Authors:** Chen Liang, Zhiyuan Yu, Rui Li, Tao Xu, Siyu Hou, Junfu Zheng, Weina Chen, Chunzeng Jia, Yan Gao, Pengji Gao, Lei Li

**Affiliations:** a Department of Gastroenterology, Beijing Jishuitan Hospital, Capital Medical University, Beijing, China; b Department of General Surgery, Beijing Friendship Hospital, Capital Medical University, Beijing, China; c School of Medicine, Nankai University, Tianjin, China; d Department of General Surgery, Beijing Jishuitan Hospital, Capital Medical University, Beijing, China

**Keywords:** immunotherapy, LAGC, meta-analysis, neoadjuvant regimens, targeted therapy

## Abstract

**Background:**

The integration of chemotherapy, targeted therapy, and immunotherapy has emerged as the latest research focus for locally advanced gastric cancer (LAGC). This study aims to evaluate the efficacy and safety of various immunotherapy-based neoadjuvant regimens for LAGC.

**Methods:**

PubMed, EmBase, and Cochrane Library databases were systematically searched based on the predefined inclusion criteria. Subsequently, the Stata software (version 17) was employed to separately integrate the outcome indicators of the single- and dual-arm studies, thereby systematically assessing the antitumor effects of three distinct immunotherapy-based regimens: neoadjuvant immunotherapy plus chemotherapy (NICT), neoadjuvant chemotherapy plus targeted therapy (NCTT), and NICT plus targeted therapy (NICTT).

**Results:**

16 high-quality Phase II prospective single-arm clinical studies and 7 dual-arm clinical studies were selected. The pooled results from the single-arm studies indicated that immune-based neoadjuvant therapy could increase the rates of pathological complete response (pCR), severe treatment-related adverse events (TRAEs), 1- and 3-year disease-free survival (DFS), as well as 1- and 3-year overall survival (OS) to 20%, 30%, 91%, 74%, 94%, and 81%, respectively. Pairwise meta-analysis indicated that both the NICT and NICTT groups achieved higher rates of pCR, major pathological response (MPR), and complete resection (R0 resection) compared to the neoadjuvant chemotherapy (NCT) group, with the NICTT group exhibiting more pronounced effects. The NICTT group exhibited a significantly higher risk of experiencing severe TRAEs compared to the NCT group. ICIs + XELOX appeared to be a more appropriate NICT regimen, as it effectively enhanced the tumor response rate while inducing less severe TRAEs. Tumors exhibiting positive expression of PD-L1 or microsatellite instable (MSI) demonstrated a higher level of responsiveness to the NICT regimen.

**Conclusions:**

Immunotherapy-based neoadjuvant regimens exhibit an enhanced potential for promoting tumor regression; however, caution is warranted due to the elevated risk of severe TRAEs.

## Introduction

Gastric cancer (GC) is a highly malignant tumor of the digestive system associated with poor prognosis. According to the most recent global cancer statistics, GC ranks within the top five in terms of incidence and mortality. In 2022, there were approximately 970 000 new cases and around 660 000 deaths globally^[1]^.Owing to its insidious onset and rapid progression, the majority of GC cases are already in the advanced stage at the time of diagnosis, which limits the therapeutic efficacy of surgery alone. Perioperative individualized treatment strategies and radical surgical resection are critical to enhancing the overall therapeutic efficacy in locally advanced gastric cancer (LAGC)^[2,3]^.

Since the MAGIC study, multidimensional treatment modalities, including chemotherapy, targeted therapy, and immunotherapy, have been successively reported to be incorporated into the perioperative treatment of LAGC, achieving promising outcomes^[4]^. Currently, perioperative chemotherapy has emerged as the standard and routine treatment for LAGC. The application of molecular targeted therapy in LAGC treatment is restricted by the significant heterogeneity observed in individual patients and tumor tissues. Tumor immunotherapy, particularly that involving immune checkpoint inhibitors (ICIs), exhibits the advantages of a low recurrence rate and high specificity, and has witnessed rapid development in recent years^[5,6]^. Multiple randomized controlled trials (RCTs) in tumor immunotherapy, such as CheckMate-649, ORIENT-16, and RATIONALE-305, have consistently demonstrated that the combination of immunotherapy with chemotherapy for patients with LAGC significantly improves survival outcomes^[7-9]^.

The successful implementation of immunotherapy in advanced GC has prompted surgeons to explore its application in neoadjuvant therapy. Although neoadjuvant immunotherapy was introduced relatively recently in LAGC, the preliminary results have been promising. Our prior research has demonstrated that incorporating immunotherapy into neoadjuvant chemotherapy (NCT) can further enhance the therapeutic efficacy, while the associated adverse reactions remain within an acceptable range^[10]^. With the evolution of treatment paradigms, a novel neoadjuvant regimen comprising the ICIs, targeted drugs, and chemotherapy has emerged and been preliminarily implemented. Currently, the investigation of neoadjuvant immunotherapy remains in its nascent stages, characterized by limitations such as a limited sample size, single-center studies, and relatively brief follow-up duration. The optimal combination for immunotherapy-based neoadjuvant regimens remains an active topic of ongoing discussion^[11]^.

## Materials and methods

### Study design and literature search

We conducted and reported this meta-analysis in accordance with the PRISMA^[12]^ and AMSTAR Guidelines^[13]^. This article is compliant with the TITAN Guidelines 2025-governing declaration and use of artificial intelligence (AI)^[14]^. PubMed, EmBase, and Cochrane Library databases were systematically searched using both subject and random terms. The search time frame was set from the inception of each database to April 2025. The scope of the search encompassed both published clinical studies and registered clinical trials. A detailed search strategy is provided in Supplemental material 1, http://links.lww.com/JS9/E475. Two reviewers independently screened titles, abstracts, and, if potentially eligible, full texts for inclusion. Disagreement was resolved by a third reviewer. Summaries for different time periods of the same clinical trial included only the most recently published version to ensure the presentation of up-to-date and relevant information.

### Inclusion criteria and data extraction

High-quality prospective single- or dual-arm cohort studies, as well as RCTs, evaluating neoadjuvant immunotherapy plus chemotherapy (NICT) or NICT plus targeted therapy (NICTT) for the treatment of LAGC were included. This study placed no restrictions on the publication language, sample size, or follow-up duration of the included studies. Additionally, subjects were required to have LAGC that remained untreated and exhibited no distant metastasis. Two researchers independently performed literature assessment and data extraction, and resolved discrepancies through discussion. The baseline characteristics to be extracted and collated encompassed the author, publication year, study type, ClinicalTrials.gov identifier, sample size, gender, age, cTNM tumor stage, intervention regimes, and treatment cycle. Outcomes that require observation and summarization included pathological complete response (pCR), major pathological response (MPR), complete resection (R0 resection), severe treatment-related adverse events (TRAEs), surgical complications, and survival outcomes. The term “severe TRAEs” was used to describe adverse events of grade 3 or higher.

**HIGHLIGHTS**

- This study conducted a comprehensive meta-analysis of published high-quality single- and dual-arm clinical trials to evaluate the efficacy of immunotherapy-based neoadjuvant regimens for LAGC.
- Immunotherapy-based neoadjuvant regimens can promote tumor regression, but increase the risk of severe TRAEs.
- Tumors exhibiting positive expression of PD-L1 or MSI demonstrate a higher level of responsiveness to the NICT regimen.
- ICIs + XELOX appears to be a more appropriate NICT regimen, as it effectively enhances the tumor response rate while inducing less severe TRAEs.

### Quality assessment

The quality assessment of single-arm clinical trials was conducted using the MINORS checklist^[15]^. Each item was assigned a score of 0 (not reported), 1 (reported but inadequate), or 2 (reported and adequate). A cumulative score of 10 or higher suggests acceptable literature quality. The tool developed by the Cochrane Collaboration was employed to evaluate the quality of RCTs^[16]^. This tool evaluates bias in six aspects, including selection, implementation, evaluation, follow-up, reporting, and other. Subsequently, the quality of RCTs was categorized as high (6 points), moderate (4-5 points), and low (0-3 points) based on the scores across the aforementioned six criteria. In addition, the quality of dual-arm cohort studies was evaluated using the Newcastle Ottawa scale (NOS). Two authors independently evaluated the quality of included studies, and any discrepancies were resolved through discussion or by consulting a third reviewer when necessary.

### Statistical analysis

All statistical analyses were conducted using Stata (version 17) software. In the single-arm and pairwise meta-analyses, the pooled effect size (ES) and odds ratio (OR), along with their respective 95% confidence intervals (CI), were estimated. The chi-squared (*χ*^2^) test and Q-test (*I*^2^) were employed to evaluate the level of heterogeneity among the incorporated studies. *I*^2^ > 50% or *P* < 0.1 was considered substantial heterogeneity, random effect model was adopted; otherwise, a fixed effect model was utilized. Subgroups were stratified based on distinct neoadjuvant treatment regimens to minimize the heterogeneity across studies. Besides, publication bias was evaluated using the Egger and Begg tests, and visualized through a funnel plot. Sensitivity analyses were performed for dual-arm studies, and the stability of pooled results was assessed by sequentially excluding each study. All statistical tests were two-sided, and *P* < 0.05 was deemed to signify statistical significance.

## Results

### Study selection and study characteristics

**Figure 1.**
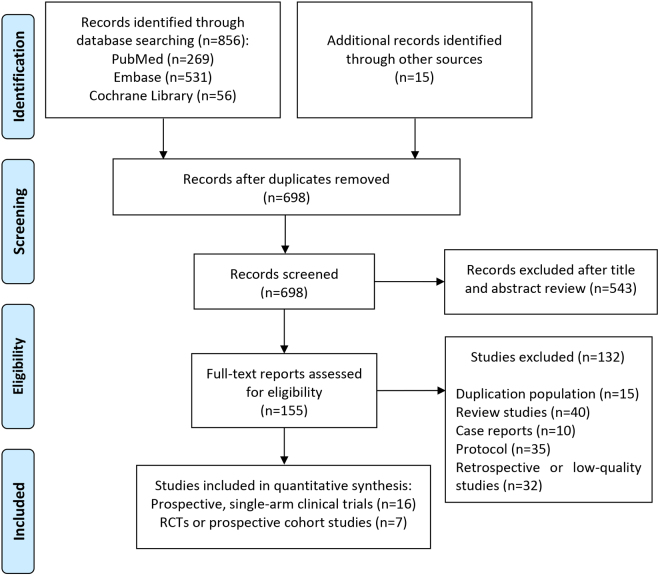
Flow chart of the studies screening and selection. RCTs, randomized controlled trials.

According to the developed strategy, a comprehensive search of the databases was conducted, identifying 856 potential studies for further evaluation. By reviewing the titles and abstracts of literature, studies that were repetitive or irrelevant were preliminarily excluded. 155 studies were selected for full-text reading and evaluation. Of these, 16 high-quality Phase II prospective single-arm clinical studies and 7 dual-arm clinical studies (6 RCTs and 1 prospective cohort trails) that fulfilled the inclusion criteria were incorporated into the meta-analysis^[17–39]^ (Fig. 1). Shitara’s study comprised two distinct intervention cohorts, namely the main cohort and the FLOT cohort. Consequently, each cohort was provided with separate descriptions and analyses^[33]^. The study quality of single-arm clinical trails and RCTs was evaluated using the MINORS and Cochrane checklists, respectively. It is important to highlight that the 23 studies included in this meta-analysis were of high quality.

### Single-arm meta-analysis

Sixteen high-quality, Phase II, prospective, single-arm clinical studies involving a total of 587 participants were selected. Among these studies, 4 evaluated the efficacy of NICTT^[17–20]^, while another 12 evaluated the efficacy of NICT in LAGC^[21–32]^. Additionally, of the 16 studies included, 14 were conducted in Asian countries^[17–25,27–29,31,32]^, with 13 originating specifically from China^[17–21,23–25,27–29,31,32]^. Baseline characteristics of the included Phase II, prospective, single-arm clinical studies are delineated in Table 1.

**Table 1:** Baseline characteristics of the included Phase II, prospective, single-arm clinical studies

	ClinicalTrials.gov identifier	Sample size	Male/female	Age/y	Tumor stage	Intervention regimes	No. of cycles	Quality score
NICTT								
Ma^[17]^ 2025	NCT03950271	25	20/5	61 (50-75)	cT_3-4a_N _+_M_0_	Camrelizumab + trastuzumab + XELOX	4	12
Chai^[18]^ 2024	ChiCTR2300075446	28	NA	NA	cT_3-4a_N _+_M_0_	Camrelizumab + disitamab vedotin + S-1	3	10
Zhou^[19]^ 2024	ChiCTR2200055269	30	26/4	58 (35-76)	cT_3-4a_N_0-3_M_0_ (stage IIB-III)	Sintilimab + apatinib + FLOT	4	12
Li^[20]^ 2022	NCT03878472	25	19/6	63 (48-70)	cT_4_N_2-3_M_0_	Camrelizumab + apatinib + SOX/S-1	≥2	11
NICT								
He^[21]^ 2025	NCT05715632	46	36/10	54 (44-64)	cT_3-4a_N _+_M_0_	Camrelizumab + XELOX	4	11
Kang^[22]^ 2025	NCT04221555	50	36/14	63 (39-83)	cT_2-4b_N_0-3_M_0_ (stage IIA-IIIC)	Durvalumab + DOS	3	12
Long^[23]^ 2025	ChiCTR2200066893	38	34/4	57 (38-74)	cT_3-4_N _+_M_0_	Cadonilimab + FLOT	3-4	11
Li^[24]^ 2024	NCT04341857	32	25/7	58 (43-70)	cT_3-4a_N _+_M_0_ (stage III)	Sintilimab + FLOT	3	12
Sun^[25]^ 2024	NCT04890392	49	39/10	58.5 (34-74)	cT_3-4a_N _+_M_0_	Tislelizumab + OX	3	12
Verschoor^[26]^ 2024	NCT03448835	21	19/2	62 (46-76)	cT_2-4a_N_0-3_M_0_ (stage IB-IIIB)	Atezolizumab + DOC	5	12
Zhao^[27]^ 2024	NCT03939962	60	43/17	58 (29-72)	cT_2-4_N _+_M_0_ (stage II-III)	Camrelizumab + mFOLFOX6	4	11
Zhong^[28]^ 2024	NCT05602935	29	18/11	62 (39-84)	cT_3-4b_N _+_M_0_ (stage III-ⅣA)	Camrelizumab + SOX	3	10
Liu^[29]^ 2023	NA	54	NA	67 (39-84)	NA	Toripalimab + FOLFIRINOX/SOX	4	10
Manji^[30]^ 2023	NCT02918162	34	23/11	65.5 (25-90)	NA	Pembrolizumab + XELOX	4	12
Jiang^[31]^ 2022	NCT04065282	36	24/12	65.5 (43-76)	cT_3-4a_N_0-3_M_0_ (stage IIB-III)	Sintilimab + XELOX	3	11
Guo^[32]^ 2022	ChiCTR2000030414	30	18/12	62 (30-72)	cT_3-4_N _+_M_0_ (stage III)	Sintilimab + XELOX	4	10

DOC, docetaxel + oxaliplatin + capecitabine; DOS, docetaxel + oxaliplatin + S-1; FLOT, fluorouracil + oxaliplatin + tetrahydrofolic acid + docetaxe; FOLFOX, oxaliplatin + calcium levofolinate + fluorouracil; FOLFIRINOX, fluorouracil + leucovorin calcium + oxaliplatin + irinotecan; NICT, neoadjuvant immunotherapy plus chemotherapy; NICTT, NICT plus targeted therapy; SOX, S-1 + oxaliplatin; XELOX, capecitabine + oxaliplatin.

#### pCR and severe TRAEs

**Figure 2.**
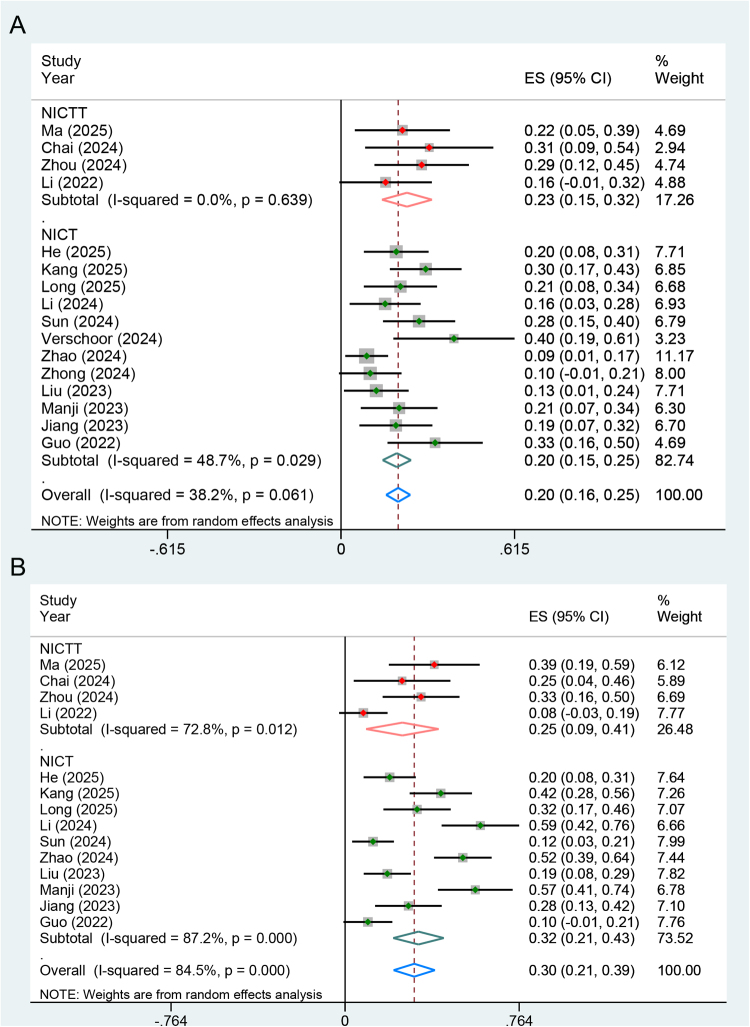
Forest plots illustrating the pooled results of (**A**) pCR and (**B**) severe TRAEs outcomes from single-arm prospective cohort studies. PCR, pathological complete response; TRAEs, treatment-related adverse events; NICT, neoadjuvant immunotherapy plus chemotherapy; NICTT, NICT plus targeted therapy; ES, effect size; CI, confidence interval.

pCR outcomes were reported in 16 studies, involving 535 participants who completed the neoadjuvant regimens and subsequently underwent evaluation. A random-effect model was employed. The pooled ES of pCR was 0.23 [95% confidence interval (CI): 0.15-0.32] in the NICTT group and 0.20 (95% CI: 0.15-0.25) in the NICT group, respectively (Fig. 2A).

14 studies reported the severe TRAEs outcomes. Four studies (94 participants) evaluated the NICTT regimes, while 10 studies (430 participants) evaluated the NICT regimes. The pooled ES of severe TRAEs was 0.25 (95% CI: 0.09-0.41) in the NICTT group and 0.32 (95% CI: 0.21-0.43) in the NICT group, respectively (Fig. 2B). Four studies reported detailed findings regarding immune-related adverse events (IrAEs). Notably, heterogeneity was observed across these studies, and the pooled ES was 0.51 (95% CI: 0.35-0.66).

#### Other outcomes

MPR outcomes were reported in 14 studies. Among these, four studies (86 participants) evaluated the NICTT regimes, while 10 studies (367 participants) evaluated the NICT regimes. The pooled ES of MPR was 0.45 (95% CI: 0.32-0.58) in the NICTT group and 0.5 (95% CI:0.39-0.61) in the NICT group, respectively (Supplemental material 2A, http://links.lww.com/JS9/E476).

Nine studies reported the surgical complications outcomes. The pooled ES of surgical complications was 0.67 (95% CI: 0.39-0.94) in the NICTT group and 0.39 (95% CI:0.17-0.61) in the NICT group, respectively (Supplemental material 2B, http://links.lww.com/JS9/E476).

**Figure 3.**
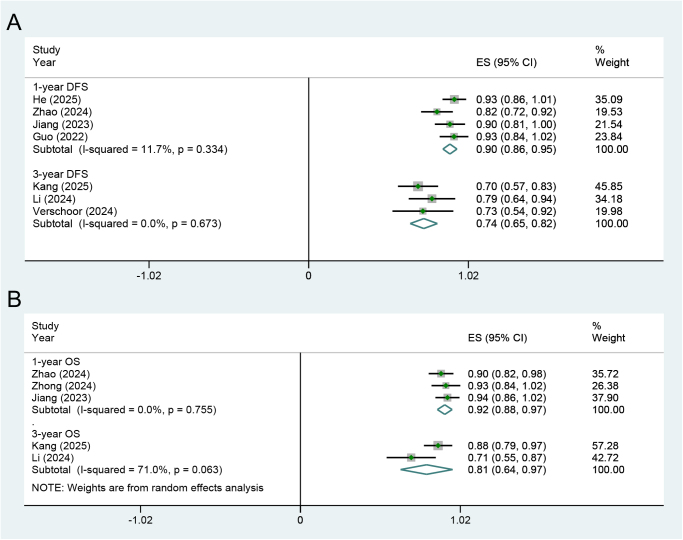
Forest plots illustrating the pooled results of (**A**) DFS and (**B**) OS rates derived from single-arm clinical studies on the NICT regimen. DFS, disease-free survival; OS, overall survival; NICT, neoadjuvant immunotherapy plus chemotherapy; ES, effect size; CI, confidence interval.

Meta-analyses were also conducted to evaluate the survival outcomes of the NICT group. The pooled ES for 1- and 3-year disease-free survival (DFS) were 0.90 (95% CI: 0.86-0.95) and 0.74 (95% CI: 0.65-0.82), respectively (Fig. 3A). Additionally, the pooled ES for 1- and 3-year overall survival (OS) were 0.92 (95% CI: 0.88-0.97) and 0.81 (95% CI: 0.64-0.97), respectively (Fig. 3B).

### Pairwise meta-analysis

Seven dual-arm clinical studies (six RCTs and one prospective cohort trails) involving a total of 1,935 participants were selected^[33–39]^. Among these studies, three compared NICT with NCT^[33–35]^, two compared NICTT with NCT^[36,37]^, and the remaining two compared NICTT with NCT plus targeted therapy (NCTT)^[38,39]^. A total of 970 and 965 LAGC patients were respectively enrolled in the immune-based neoadjuvant therapy group and the control group. Baseline characteristics of the included RCTs and prospective cohort studies are delineated in Table 2.

**Table 2:** Baseline characteristics of the included RCTs and prospective cohort studies

Study (Ref.) year	Study type	Sample size	Male/ Female	Age/y	Tumor stage	Intervention regimes	Control regimes	No. of cycles	Quality score
NICT vs. NCT									
Shitara^[33]^ 2024	Phase III RCT	402/402	575/229	64/63^a^	cT_1-4_N_0-3_M_0_ (stage II-IVA)	Pembrolizumab + control regimens	Cisplatin + capecitabine/fluorouracil	3	6
Shitara^[33]^ 2024	Phase III RCT	100/103	NA	NA	NA	Pembrolizumab + FLOT	FLOT	4	6
Lorenzen^[34]^ 2023	Phase II/III RCT	146/149	217/78	61/62^a^	cT_1-4_N_0-3_M_0_	Atezolizumab + FLOT	FLOT	4	4
Min^[35]^ 2022	RCT	31/23	42/12	63/63^a^	NA	Camrelizumab + FLOT	FLOT	4	4
NICTT vs. NCT									
Lin^[36]^ 2024	Phase II RCT	51/53	77/27	63/63^a^	cT_3-4_N _+_M_0_	Camrelizumab + apatinib + SAP	SAP	3	4
Li^[37]^ 2024	Phase III RCT	180/180	296/64	63/63^a^	cT_3-4b_N _+_M_0_	Camrelizumab + rivoceranib + SOX	SOX	3	6
NICTT vs. NCTT									
Peng^[38]^ 2024	Phase II RCT	21/21	39/3	61/65^a^	NA	Atezolizumab + control regimens	Trastuzumab + XELOX	3	4
Wang^[39]^ 2023	prospective cohort	39/34	50/23	57.9/ 58.9^b^	cT_3-4a_N _+_M_0_ (stage III)	Sintilimab/carrelizumab/toripalimab + control regimens	Apatinib + SOX/XELOX	3	6

FLOT, fluorouracil + oxaliplatin + tetrahydrofolic acid + docetaxe; NCT, neoadjuvant chemotherapy; NCTT, NCT plus targeted therapy; NICT, neoadjuvant immunotherapy plus chemotherapy; NICTT, NICT plus targeted therapy; RCT, randomized controlled trial; SAP, S-1 + nab-paclitaxel; SOX, S-1 + oxaliplatin; XELOX, capecitabine + oxaliplatin.

^a^ Values are presented as median.

^b^ Values are presented as mean.

#### pCR, MPR, and R0 resection

**Figure 4.**
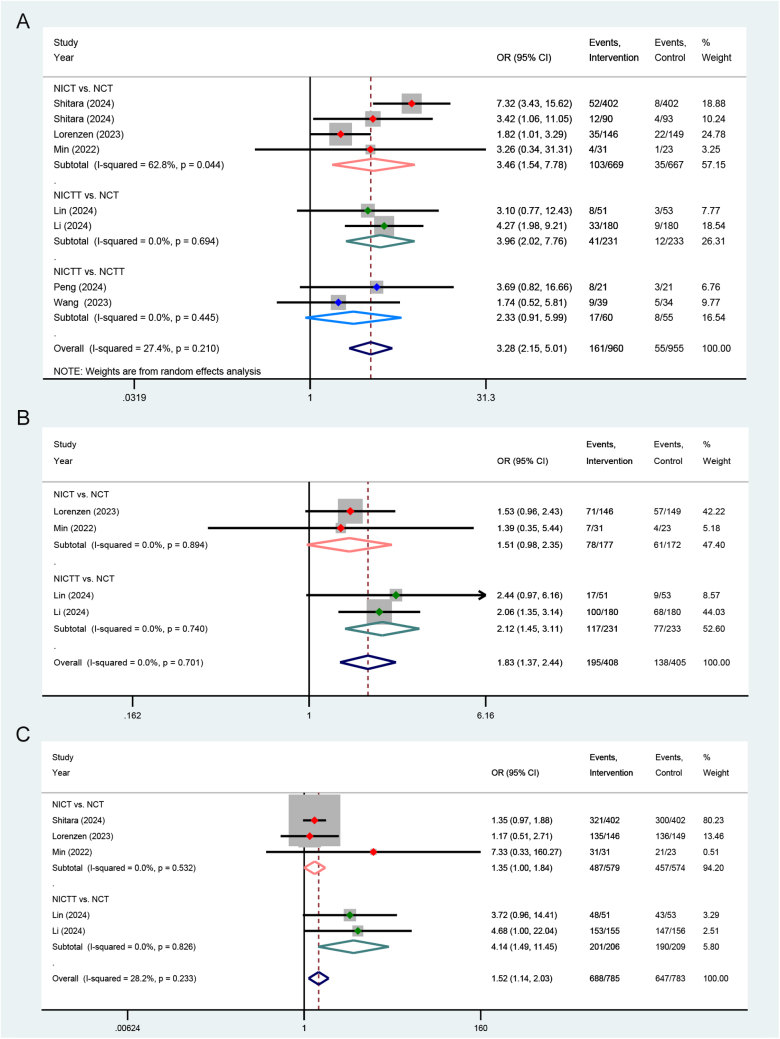
Forest plots illustrating the pooled results of (**A**) pCR, (**B**) MPR, and (**C**) R0 resection outcomes from RCTs and dual-arm prospective cohort studies. PCR, pathological complete response; MPR, major pathological response; RCTs, randomized controlled trials; NCT, neoadjuvant chemotherapy; NCTT, NCT plus targeted therapy; NICT, neoadjuvant immunotherapy plus chemotherapy; NICTT, NICT plus targeted therapy; OR, odds ratio; CI, confidence interval.

All the included studies reported the pCR outcomes. Inclusion studies comparing NICT and NCT demonstrated heterogeneity (*I*^2^ = 62.8%, *P* = 0.044). Consequently, a random-effect model was employed for the meta-analysis. The pooled results demonstrated that, in comparison to the NCT group, both the NICT (OR = 3.46, *P* = 0.003) and NICTT groups (OR = 3.96, *P*<0.001) exhibited a higher rate of pCR. NICTT group demonstrated a higher tendency toward pCR compared to the NCTT group (OR = 2.33, *P* = 0.078). Above all, the immune-based neoadjuvant therapy group demonstrated a superior ability to achieve pCR (OR = 3.28, *P*<0.001) (Fig. 4A).

MPR and R0 resection outcomes were reported in four and five studies, respectively. Since there was no significant heterogeneity observed within each subgroup, fixed-effect models were employed. The pooled results demonstrated that the NICTT group achieved a higher MPR (OR = 2.12, *P*<0.001) and R0 resection rates (OR = 4.14, *P* = 0.006) compared to the NCT group. Additionally, the NICT group exhibited a tendency toward higher MPR (OR = 1.51, *P* = 0.065) and R0 resection rates (OR = 1.35, *P* = 0.051) compared to the NCT group. Above all, the immune-based neoadjuvant therapy group demonstrated a superior ability to achieve MPR (OR = 1.83, *P*<0.001) (Fig. 4B) and R0 resection (OR = 1.52, *P* = 0.005) (Fig. 4C).

#### Severe TRAEs

**Figure 5.**
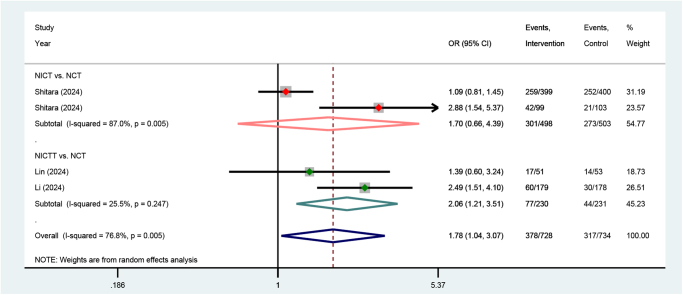
Forest plots illustrating the pooled results of severe TRAEs outcomes from RCTs and dual-arm prospective cohort studies. TRAEs, treatment-related adverse events; NCT, neoadjuvant chemotherapy; NICT, neoadjuvant immunotherapy plus chemotherapy; NICTT, NICT plus targeted therapy; OR, odds ratio; CI, confidence interval.

Three studies reported the severe TRAEs outcomes. One of the studies comprised two cohorts, each of which was analyzed as a distinct group in the meta-analysis. Pooled results from random-effect model demonstrated a higher incidence of severe TRAEs in the NICTT group compared to the NCT group (OR = 2.06, *P* = 0.007), whereas no significant increase in risk was observed in the NICT group (OR = 1.70, *P* = 0.276) (Fig. 5).

### Subgroup analysis

**Figure 6.**
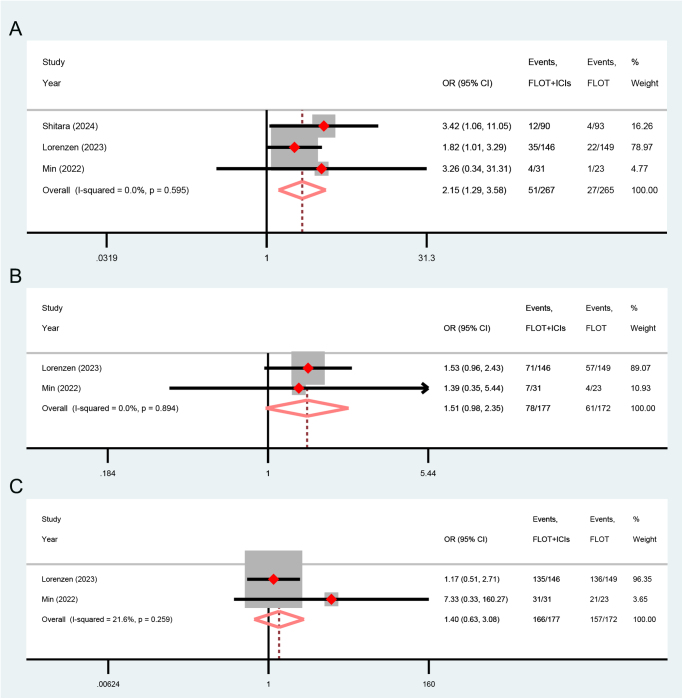
Forest plots illustrating the subgroup analysis outcomes for (**A**) pCR, (**B**) MPR, and (**C**) severe TRAEs derived from the dual-arm studies of specific chemotherapy drug regimens. PCR, pathological complete response; MPR, major pathological response; TRAEs, treatment-related adverse events; OR, odds ratio; CI, confidence interval; FLOT, docetaxel + oxaliplatin + tetrahydrofolic acid + fluorouracil; ICIs, immune checkpoint inhibitors.

We further stratified the primary outcome indicators of the meta-analysis based on distinct neoadjuvant medication regimens and performed subgroup analyses accordingly. The results of the subgroup analysis for the single-arm studies are presented in Table 3. The integration of ICIs into XELOX regimen appeared to enhance the tumor response more effectively while being associated with fewer severe TRAEs. In addition, the subgroup analysis for dual-arm studies revealed that integrating ICIs into the FLOT regimen was associated with an increased probability of achieving pCR (OR = 2.15, *P* = 0.003) (Fig. 6A) and MPR (OR = 1.51, *P* = 0.065) (Fig. 6B), while maintaining a comparable risk profile for severe TRAEs (OR = 1.40, *P* = 0.406) (Fig. 6C).

**Table 3:** Subgroup analysis of the primary outcome measures in the single-arm studies stratified by different neoadjuvant regimens

Number of studies	Neoadjuvant regimens	Number of events	Number of patients	Type of effect model	Tests of heterogeneity	Pooled ES (95% CI)
pCR						
4	XELOX + ICIs	33	146	Fixed	*I*^2^ = 0%, *P* = 0.552	0.22 (0.15, 0.29)
2	FLOT + ICIs	13	70	Fixed	*I*^2^ = 0%, *P* = 0.556	0.18 (0.09, 0.27)
2	SOX + ICIs	16	76	Random	*I*^2^ = 75.1%, *P* = 0.045	0.19 (0.02, 0.36)
MPR						
4	XELOX + ICIs	74	146	Fixed	*I*^2^ = 36.4%, *P* = 0.193	0.51 (0.43, 0.59)
2	FLOT + ICIs	33	70	Fixed	*I*^2^ = 0%, *P* = 0.660	0.47 (0.35, 0.59)
2	SOX + ICIs	44	76	Random	*I*^2^ = 60.4%, *P* = 0.112	0.59 (0.42, 0.77)
Severe TRAEs					
4	XELOX + ICIs	42	147	Random	*I*^2^ = 87.0%, *P* < 0.001	0.28 (0.10, 0.46)
2	FLOT + ICIs	31	70	Random	*I*^2^ = 82.9%, *P* = 0.016	0.45 (0.18, 0.72)

CI, confidence intervals; ES, effect size; FLOT, docetaxel + oxaliplatin + tetrahydrofolic acid + fluorouracil; ICIs, immune checkpoint inhibitors; MPR, major pathological response; pCR, pathological complete response; SOX, S-1 + oxaliplatin; TRAEs, treatment-related adverse events; XELOX, capecitabine + oxaliplatin.

**Figure 7.**
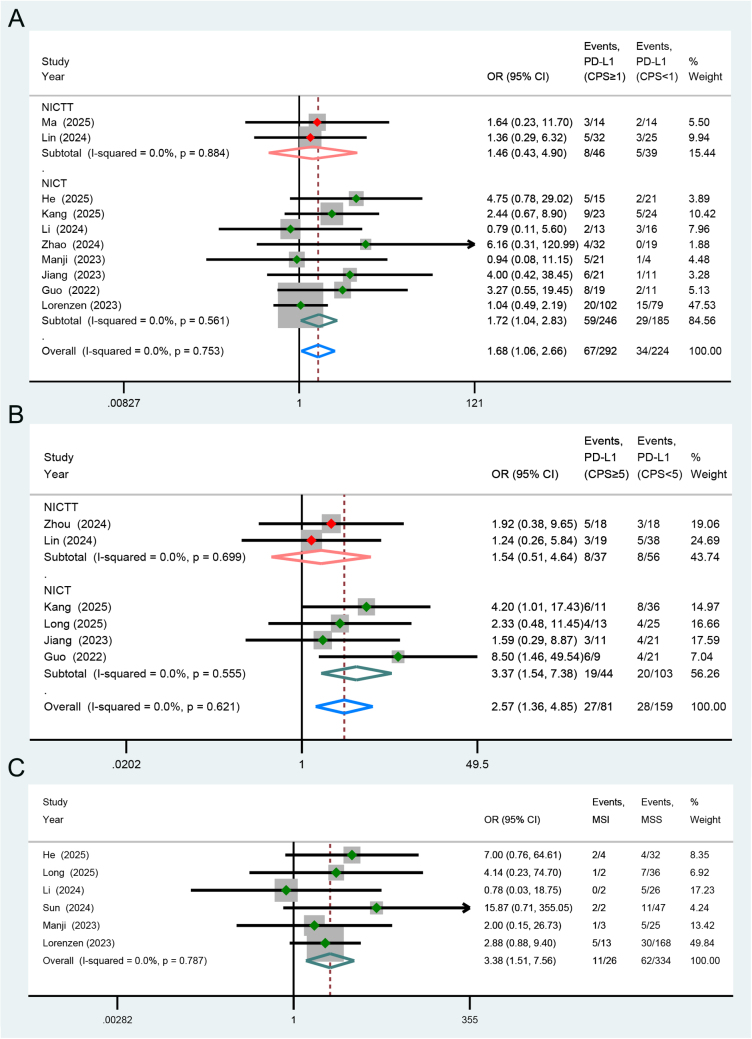
Forest plots illustrating the impact of (**A**) PD-L1 CPS≥1, (**B**) CPS≥5, and (**C**) MSI on the pCR outcomes following the NICT and NICTT regimens. CPS, combined positive score; MSI, microsatellite instable; MSS, microsatellite stable; pCR, pathological complete response; NICT, neoadjuvant immunotherapy plus chemotherapy; NICTT, NICT plus targeted therapy; OR, odds ratio; CI, confidence interval.

In addition, we categorized the cases in the NICT and NICTT groups according to the PD-L1 expression levels or microsatellite stability status of the tumors, and systematically compared and summarized the pCR outcomes between the groups. Whether PD-L1 positive expression was defined by combined positive score (CPS) > 1 (OR = 1.72, *P* = 0.034) (Fig. 7A) or CPS > 5 (OR = 3.37, *P* = 0.002) (Fig. 7B), the positive group demonstrated a higher response rate to NICT and exhibited a greater likelihood of achieving pCR. Compared to microsatellite stable (MSS) tumors, microsatellite instable (MSI) tumors were more likely to achieve pCR following NICT regimes (OR = 3.38, *P* = 0.003) (Fig. 7C).

### Publication bias and sensitivity analysis

**Figure 8.**
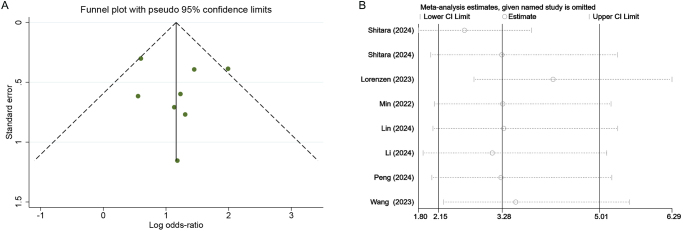
(**A**) Funnel plot and (**B**) sensitivity analysis were constructed based on pCR outcomes derived from dual-arm clinical studies. PCR, pathological complete response.

By reviewing relevant literature and consulting with statistical experts, it has been determined that conducting publication bias tests or sensitivity analyses for the meta-analysis of single-arm studies holds limited significance. Since the single-group rate serves merely as a descriptive statistic rather than a result from a difference comparison, it is inappropriate to characterize it as either “positive” or statistically significant. Therefore, we exclusively performed publication bias and sensitivity analyses for the paired meta-analysis. The Egger (*P* = 0.830) or Begg test (*P* = 0.902), conducted based on the pCR outcomes of the dual-arm studies, did not demonstrate statistically significant differences. The publication bias was also evaluated by constructing funnel plot for pCR outcomes derived from dual-arm studies (Fig. 8A). The aforementioned analysis indicated that the possibility of publication bias in the dual-arm studies was relatively low. Additionally, sensitivity analysis was conducted for pCR outcomes derived from dual-arm studies (Fig. 8B), and no significant differences were observed after systematically excluding each study, which confirmed the stability of our findings.

## Discussion

In recent years, with the accumulation of clinical research evidence and experience, as well as the continuous evolution of treatment concepts, the neoadjuvant treatment model for LAGC has entered a new phase. Immune escape is one of the critical mechanisms underlying malignant tumor progression. Programmed cell death protein 1 (PD-1), a molecule primarily expressed on the surface of T cells, B cells, and myeloid cells, serves an inhibitory and regulatory function within the immune system. Consequently, targeted inhibition of this molecule can restore the immune system’s ability to effectively monitor, recognize, and eliminate tumor cells^[11,40,41]^. Previous studies have successfully demonstrated the application of PD-1 inhibitors in combination with other therapies for advanced GC, thereby establishing the feasibility of a novel neoadjuvant regimen that combines PD-1 inhibitors with chemotherapy and/or targeted drugs. Currently, there is a paucity of clinical practice and research investigating the application of PD-1 inhibitors in neoadjuvant therapy, particularly within high-quality multicenter RCTs^[7,42,43]^. Consequently, we conducted a comprehensive meta-analysis of published high-quality single- and dual-arm clinical trials to evaluate the efficacy of immunotherapy-based neoadjuvant regimens for LAGC, aiming to provide robust, high-level evidence to guide clinical practice. Compared with published studies of the same type, the analysis process in this study was more intricate, and the research outcomes yield richer findings.

The meta-analysis of single-arm studies demonstrated that NICT and NICTT could significantly enhance the pCR rate for LAGC to 20% and 23%, respectively. Pairwise meta-analysis also indicated that both the NICT and NICTT groups achieved higher rates of pCR, MPR, and R0 resection compared to the NCT group. Numerous studies have demonstrated that NCT effectively reduces tumor staging and enhances the resection rate, thereby establishing it as a conventional treatment strategy for LAGC^[2-5,44]^. ICIs, which constitute a pivotal component of immunotherapy, employ monoclonal antibodies to specifically bind to immune checkpoints present on the surface of tumors or immune cells. This binding blocks negative regulation, reactivates the immune system, and effectively eradicates tumor cells. The emergence and application of ICIs have fundamentally transformed the conventional treatment approaches for various malignant solid tumors, including LAGC^[5,6,10,11]^. Combining chemotherapy with immunotherapy can achieve synergistic effects that enhance the anti-cancer response. On the one hand, the combination of chemotherapy and immunotherapy can augment the immune response against tumors, thereby enhancing patient sensitivity to treatment and improving the response rate. On the other hand, this combination facilitates the activation of the immune system to recognize and target tumor stem cells, thus promoting tumor regression and improving the durability of therapeutic effects^[45,46]^. Targeted drugs are capable of modulating the immunosuppressive characteristics of the tumor microenvironment (TME) through specific interaction with key signaling pathways in tumor cells or stromal cells. ICIs and chimeric antigen receptor T-cell (CAR-T) therapy have demonstrated the ability to eliminate tumor cells by alleviating T-cell inhibition or through direct targeting mechanisms. The essence of the synergistic effect of targeted immunotherapy lies in “modulating” the tumor microenvironment using targeted drugs and “enhancing” the sustained immune response through immunotherapy. The aforementioned integrated strategies exemplify the profound convergence of precision medicine and immune regulation, offering novel insights into addressing tumor heterogeneity and drug resistance^[36–39]^.

Drug toxicity and side effects have consistently posed significant challenges for scholars in the field of oncology. Consequently, TRAEs associated with chemotherapy, targeted therapy, and immunotherapy during neoadjuvant treatment for LAGC remain a focal point of attention. In this study, the incidence of severe TRAEs in immunotherapy-based neoadjuvant group exceeded 25%, a significantly higher rate compared to the NCT group. As a critical component of TRAEs, our study also demonstrated that the incidence of IrAEs in the NICT group can reach up to 50%. IrAEs is believed to result from impaired self-tolerance due to the loss of T-cell inhibition and can manifest across all organ systems. According to previous studies, common IrAEs include reactive cutaneous capillary endothelial proliferation, hepatitis, headache, hyperthyroidism, and diarrhea, among others^[21,26,28,30]^. Immunotherapy drugs may decrease patients’ tolerance to other medications, exacerbate bone marrow suppression, induce gastrointestinal complications, and adversely affect vital organ functions, including the heart, lungs, adrenal glands, and thyroid. When the aforementioned issues are collectively considered, they can substantially compromise the overall health of the patients, resulting in a marked decrease in surgical tolerance, an elevated risk of perioperative complications, and prolonged postoperative recovery^[19,20,47]^. The mainstay of treatment for IrAEs involves the use of corticosteroids or other immunosuppressive agents. With appropriate management, the majority of irAEs can be effectively controlled. However, some severe cases of IrAEs may prove fatal. Careful management coupled with effective interventions can enhance patient outcomes, ensuring that TRAEs are minimized while maximizing the survival benefits associated with increased pCR and MPR rates. Therefore, it is imperative to closely monitor the patient’s physiological responses during immunotherapy and adjust the treatment regimen as per the specific circumstances to ensure optimal therapeutic balance. Establish an effective correlation between chemotherapy and surgery. When necessary, consider delaying the operation schedule to ensure optimal surgical outcomes and minimize associated risks^[11,47,48]^.

Our meta-analysis demonstrated that DFS rates at 1- and 3-year post-surgery in the NICT group was 90% and 74%, respectively, while OS rates at the same time points was 92% and 81%, respectively. Given the limited research within the NICTT group and the fact that the survival outcomes pooled at each time point were derived from non-overlapping studies, the reliability and consistency of the meta-analysis results may be compromised. An interim analysis of the multicenter RCT study demonstrated that the median event-free survival in the NICT (pembrolizumab plus cisplatin-based chemotherapy) group (44.4 months, 95% CI 33.0-not reached) was longer than that in the NCT group (25.3 months, 95% CI 20.6-33.9). However, the comparison of median OS between the NICT (60.7 months, 95% CI 51.5-not reached) and NCT (58.0 months, 95% CI 41.5-not reached) groups did not reach statistical significance^[33]^. Most of the outcome data from existing relevant RCT studies were derived from the preliminary and interim results of Phase II/III clinical trials, which had limited follow-up durations or lacked reported survival outcomes. Consequently, it was challenging to conduct effective pooled analyses. The long-term survival benefits of immunotherapy-based neoadjuvant regimens for LAGC patients remain to be confirmed by further follow-up studies.

Human epidermal growth factor receptor 2 (HER-2) and vascular endothelial growth factor receptor (VEGFR) are the most frequently targeted molecules in LAGC therapy. Trastuzumab and Apatinib, inhibitors targeting the aforementioned targets, have been extensively validated in clinical studies to effectively synergize with chemotherapy, thereby enhancing the therapeutic efficacy for LAGC^[49,50]^. The successful implementation of the NICT and NCTT regimens has prompted scholars to shift their research focus towards the NICTT triple regimen. KEYNOTE-811 study has confirmed that the addition of ICIs to anti-HER-2 therapy plus chemotherapy can significantly enhance the objective response rate, DFS, and OS^[51]^. NCT03192735 study also confirmed the efficacy and acceptable safety profile of apatinib in combination with SOX as a neoadjuvant treatment for LAGC^[52]^. The results of this meta-analysis indicated that the incorporation of targeted drugs in addition to NICT may facilitate tumor regression. However, this approach was also associated with an increased risk of TRAEs and complications. According to previous literature, patients who were HER2-positive (HER2+) may derive greater benefit from the NICTT regimen^[17,18,38]^. Further high-quality, long-term follow-up clinical studies are warranted to investigate the optimal combination of immunotherapy-based neoadjuvant regimens, identify the appropriate patient population, and determine the ideal treatment timing.

Although this meta-analysis involved pooling and analyzing data from the most recent and high-quality clinical studies, inherent limitations still exist. Firstly, given that single-arm studies are unable to directly compare the effectiveness of different treatment regimens, the variations in initiation time, institutions, and participant populations across studies result in substantial heterogeneity, thereby significantly undermining the reliability of pooled results. In addition, given the limited number of existing studies, particularly dual-arm studies, we could only conduct subgroup analyses based on specific chemotherapy drug regimens and could not provide summaries for specific ICIs or targeted therapies. Last but not least, given that China accounts for approximately 40% of the global GC cases, the majority of the studies included in this analysis originate from China (78%). This geographical concentration may limit the universality and generalizability of the findings to some extent.

## Conclusions

The application of immunotherapy-based neoadjuvant regimens can increase the rates of pCR, severe TRAEs, 1-, and 3-year DFS to 20%, 30%, 90%, and 74%, respectively. Compared with NCT alone, the addition of ICIs and/or targeted drugs exhibited a significantly enhanced potential for tumor regression and R0 resection. However, it is crucial to remain vigilant regarding the occurrence of severe TRAEs and surgical complications. Tumors exhibiting positive expression of PD-L1 or MSI demonstrated a higher level of responsiveness to the NICT regimen. ICIs + XELOX appeared to be a more appropriate NICT regimen, as it effectively enhanced the tumor response rate while inducing less severe TRAEs. Whether the addition of targeted drugs to NICT can further enhance the anti-tumor effect while ensuring safety requires further investigation. In light of the relatively high risk of developing severe TRAEs, we recommend selecting neoadjuvant drug combination regimens based on the expression levels of biomarkers such as PD-L1 and HER-2. For patients exhibiting positive expression of the aforementioned biomarkers, provided their physical condition allows, they may be considered as the preferentially recommended population for ICIs and targeted drugs, both in the preoperative and postoperative settings. The currently available high-quality clinical studies on neoadjuvant immunotherapy for LAGC are predominantly single-arm trials, making it challenging to directly compare the efficacy of different neoadjuvant regimens. In the future, high-quality multi-center RCT studies, which focus on specific tumor pathological characteristics and neoadjuvant medication regimens, will be essential to validate and extend the findings of this study.

## Data Availability

All data generated or analyzed and software used during this study are included in the article/Supplementary Material. Further inquiries can be directed to the corresponding author.

## Research registration unique identifying number (UIN)

PROSPERO database (CRD420251033521).
